# Pain Neuroscience Education on Reducing Opioid Dependency in African American and Caucasian Populations: A Narrative Review

**DOI:** 10.3390/jcm14124360

**Published:** 2025-06-19

**Authors:** Austin Granger, Ersilia Mirabelli

**Affiliations:** 1 Macon Rehabilitation and Performance, Macon, GA 31210, USA; 2 Department of Biology and Chemistry, School of Health Sciences, Liberty University, Lynchburg, VA 24515, USA; emirabelli@liberty.edu

**Keywords:** pain neuroscience education, opioids, ethnocultural, neuroplasticity, nociplastic pain, opioid use disorder, chronic pain

## Abstract

This review explores pain neuroscience education (PNE) in the context of opioid dependence among Caucasian and African American populations, addressing disparities and sociocultural influences in the opioid epidemic. Von Bertalanffy’s general systems theory and Bronfenbrenner’s ecological systems theory comprise the underlying theoretical frameworks behind the review, emphasizing the importance of biopsychosocial perspectives of chronic pain and ecological systems on individual development. Within these frameworks, the study objective is to summarize relevant and contemporary literature among African American and Caucasian populations regarding opioid dependency, neuroplasticity in chronic pain, and PNE. Peer-reviewed articles published within the last 10 years were reviewed for relevance. Limitations include a lack of research on the intersection of ethnicity and PNE, a lack of studies investigating interdisciplinary input regarding PNE, and a focus on only two ethnic groups. This narrative review finds that African Americans face systemic barriers to effective treatment for pain and opioid use disorder (OUD), while Caucasians are more likely to be overprescribed with higher rates of OUD. From a systems and ecological perspective, maladaptive neuroplasticity in chronic pain (biologic subsystem) intersects with ethnic disparities in prescribing access and pain beliefs (psychosocial subsystem) to influence opioid use and the chronic pain experience. PNE shows promise as an adjunct to traditional physical therapy in reducing nociplastic pain, potentially affecting opioid dependency. Future research should incorporate readiness-to-change models, generational and ethnocultural perspectives, and neuroimaging with PNE to optimize the delivery of PNE to individuals of different backgrounds.

## 1. Introduction

This literature review seeks to understand the role of pain neuroscience education (PNE) in reducing opioid dependence in Caucasian and African American patients in a physical therapy setting. Opioids have significant analgesic effects and are used in various severe chronic pain pathologies; however, they have a controversial role secondary to a high risk of dependency [1]. The opioid epidemic is an American public health crisis, resulting in nearly 400,000 deaths in the last two decades [2]. Approximately 50 million Americans experience significant morbidity and reduced quality of life secondary to opioid use disorder (OUD), resulting in annual healthcare costs of up to 600 billion dollars [3,4]. OUD is specifically high in Caucasians and African Americans. While more Caucasians historically exhibit higher rates of OUD, African Americans are experiencing disproportionately higher opioid overdose rates, specifically in the context of polysubstance use [5,6]. Subsequently, a notable difference in care between these two populations is noted in contemporary literature, with variation in medication quantity and quality in the management of OUD and chronic pain [7,8,9,10,11,12,13]. Cultural and individual beliefs about pain and self-management compound with societal influences to manifest discrepancies in the motive and method of opioid abuse [4,14,15,16,17]. For instance, more African Americans may consume opioids to self-treat versus recreational use secondary to differences in care and perceived biases in healthcare. This may be a facilitatory factor for polysubstance use, highlighting sociocultural influences as a potential explanation behind the rapid increase in fatality [5,6,18]. Many of those with OUD suffer from chronic pain, with contemporary research noting a positive correlation between pain severity and opioid dependency [19]. Nociplastic pain is a significant component of the chronic pain experience for many, and contemporary physical therapy practice has made significant headway in understanding and treating nociplastic pain conditions. One treatment methodology is PNE, which has gained significant popularity over the last two decades. In the presence of both personal and societal strain secondary to the opioid epidemic, effective utilization of PNE offers a potential strategy for reducing patient opioid dependency [20]. However, clinicians and researchers debate the effectiveness of PNE across various clinical and ethnic populations, highlighting a significant gap in contemporary literature [21,22].

## 2. Theoretical Frameworks

The literature review utilizes two theoretical frameworks to guide a systematic approach to holistic healthcare and ethnocultural implications on human development. Moseley and colleagues [23] provided an extensive review of PNE using learning frameworks; therefore, this study adds to the existing literature by integrating ethnocultural factors within a biopsychosocial lens through a systems and ecological approach. First, this section includes the background and implications of the general systems theory developed by von Bertalanffy [24], elucidating the intricacies of contemporary healthcare and the potential pitfalls of managing complex problems through a single perspective or discipline. Second, Bronfenbrenner’s ecological systems theory [25] clarifies the complex and interconnected environmental influences on individual development. Together, these two systems provide a practical framework for reviewing the opioid epidemic, PNE, and ethnocultural perspectives of pain and addiction.

### 2.1. General Systems Theory

In 1950, Ludwig von Bertalanffy introduced general systems theory as a conceptual framework for understanding the interrelationships among systems, countering reductionist ideologies of his era and noting isomorphic laws in varying disciplines. He believed that the lack of unification of the sciences regarding classification was a significant problem, resulting in studying principles or problems through individual, isolated elements rather than holistically. Subsequently, he highlighted the importance of wholeness in system complexity, emphasizing that a system is composed not only of various individual components but also of their interactions; therefore, a proper understanding of a principle or problem should emphasize a more holistic, multidisciplinary view secondary to the interdependence of the underlying elements [24].

The general systems theory is fundamental to contemporary clinical models in all healthcare disciplines. It facilitated the development of Engel’s biopsychosocial model [26], which was transformative for biomedically centered healthcare ideologies of the era. The model emphasizes that patient presentation analysis should integrate the interdependence of their psychological, sociological, and biological systems, highlighting Bertalanffy’s construct of wholeness [24,26]. Unfortunately, biomedical perspectives are markedly dominant among many patients and healthcare providers, neglecting the potential impact of other systems and resulting in faulty pain cognitions [20,27,28,29]. Understanding the above theory and model is vital to the practical, individualistic implementation of PNE.

### 2.2. Ecological Systems Theory

Developed in 1979, Bronfenbrenner’s ecological systems theory offers a framework for understanding how various systems interact dynamically to influence individual development. These systems include the microsystem (immediate environments), the mesosystem (interactions between different microsystems), the exosystem (indirect environments), and the macrosystem (cultural and societal contexts). The systems are hierarchical, meaning that larger, broader systems directly influence smaller, more local systems. His theory of dynamic interplay between direct and indirect systems influences individual development and explicitly highlights the effect of culturalism [25].

The ecological systems theory provides a fundamental framework for reviewing research regarding ethnocultural perspectives of pain and addiction. It highlights the complexities of opioid dependency across various ethnicities and cultural groups, as an individual’s beliefs may be profoundly influenced by his or her surrounding environment and temporal context. Individuals from diverse ethnic backgrounds exhibit significantly different systemic structures influenced by their cultural frameworks, spiritual beliefs, health and wellness perspectives, socioeconomic priorities, and more [25]. Understanding the interconnectedness of these underlying systems within the ecological systems theory offers a solid foundation for examining opioid dependency through the lens of an individual’s direct and indirect environments. This approach ultimately enhances ethnocultural understanding, potentially improving the efficacy of PNE intervention.

## 3. Materials and Methods

A literature review was performed by A.G. between October 19, 2024 and June 1, 2025 in databases in Jerry Falwell Library, Mercer University Library, PubMed, and Google Scholar. Peer-reviewed articles relevant to this review’s objectives and those published in the last 10 years comprised the study’s inclusion criteria. Search terms and key concepts include “pain neuroscience education”, “physical therapy”, “opioid dependence”, “opioid use disorder”, “chronic pain”, “central sensitization”, “nociplastic pain”, “neuroplasticity and pain”, “pain neuromatrix”, “peripheral processing mechanisms”, “central processing mechanisms”, “ion channel expression”, “addiction”, “ethnic perspectives of pain”, and “graded motor imagery”. A snowballing search methodology was implemented within articles identified by the above search terms, and the study included a notable variety of articles, including quantitative and qualitative study designs in addition to other review articles. The study aims to provide a summary of the existing literature and elucidate research opportunities in the growing field of PNE. The resulting research objectives include synthesizing the contemporary research regarding the opioid epidemic, the role of nociplastic pain mechanisms in chronic pain, the relationship of pain and addiction, the role of ethnicity on perceptions of pain and addiction, the effectiveness of PNE, and the clinical implementation of PNE.

This review has limitations. First, there is a paucity of research investigating the intersection between ethnicity and PNE. Although a lack of research investigating ethnicity and pain neuroscience provides ample opportunity for study, it poses a challenge for the literature review regarding comprehensiveness [21]. Additionally, a lack of interdisciplinary research exists regarding PNE implementation; therefore, the implementation of PNE may lack input from specialties other than physical therapy, narrowing the scope of the review [30]. Only two ethnic groups (African American and Caucasian) were included, which may result in a lack of applicability to other ethnicities. Inclusion of two ethnicities allows for a more detailed approach into ethnocultural differences among two of the most significantly affected populations regarding OUD [31,32,33]. Also, this article cannot make causal claims, as no statistical analysis or experimentation is performed.

## 4. Review of the Literature

This section synthesizes relevant literature to elucidate the contemporary understanding surrounding the opioid epidemic and PNE in African American and Caucasian populations. The opioid epidemic is investigated through research on opioid dependency, opioid prescription, and current medical treatment. A review of opioid use, access, and management leads to further investigation on nociplastic pain considerations in chronic pain conditions, specifically neuroplastic changes in the transition from acute to chronic pain, the pain neuromatrix, the neurophysiologic similarities between pain and addiction, and ethnic perspectives of pain. Integrating nociplastic pain research into the management of opioid dependency leads to PNE. Research regarding PNE delivery strategies and indicators for success is summarized to elucidate PNE’s potential as an effective management strategy for opioid dependency in African American and Caucasian populations. This narrative review synthesizes contemporary literature within a systems and ecological approach, investigating opioid dependency, nociplastic pain mechanisms, and the role of PNE to address the interrelationships of biological, psychological, and sociological domains and highlight the dynamic interplay of individual, interpersonal, and societal factors that influence pain management and perception.

### 4.1. Opioid Dependency

The opioid epidemic results in tremendous individual and societal strain in the United States, affecting more than 50 million Americans and resulting in over 600 billion dollars in annual healthcare costs [3,4]. This impact is reportedly larger on African Americans and Caucasians, making these populations the primary emphasis of this review [31,32,33]. Opioids have roles in the healthcare system for treating numerous types of pain, including acute, cancer, and end-of-life pain. When used for pain that is chronic and not related to cancer, there may be an increased risk of opioid misuse followed by abuse [34]. Opioid abuse can lead to OUD, a chronic disorder with significant implications for physical, social, and mental health. Personal biopsychosocial morbidity is further complicated by the stigma surrounding opioid use, amplifying the potential burden to the individual [35]. Per the ecological systems theory, an individual’s direct and indirect environment has significant influence over human development, so an understanding of ethnocultural perspectives regarding Caucasian and African American populations is beneficial in elucidating the impact of OUD on the study’s target populations [25].

#### 4.1.1. Differences in Opioid Prescription and OUD Among Caucasian and African American Populations

Chronic pain management often involves the prescription of opioid medications, and the patterns of opioid prescribing and the prevalence of OUD differ based on ethnicity. Contemporary research notes that the initial prescription rate between Caucasians and African Americans does not differ significantly; however, Caucasians are much more likely to receive a higher number of prescriptions and have a higher susceptibility to developing OUD [4,12,31,32,33]. Johnson-Jennings and colleagues [10] provide greater context to this claim by clarifying that African American patients are likely undertreated for pain complaints, potentially secondary to provider perceptions. African Americans are also more likely to receive less opioid pain medication despite reporting higher pain levels than Caucasians [7,11,12,13,36]. Nevertheless, the higher prescription rates and increased prevalence of OUD among Caucasians may be contributing factors to the 225% increase in opioid-related mortality between 1990 and 2010. This is notably different compared to opioid-related mortality in African Americans within the same timeframe, which decreased by 3% [31]. However, African American and Caucasian mortality regarding polysubstance use (e.g., opioid drugs plus cocaine) has risen 135% and 33%, respectively, between 2013 and 2018 [5]. The majority of African Americans misusing opioids are likely doing so to self-treat chronic pain symptoms, which may be facilitated by a lack of access to prescription buprenorphine [18,36,37,38,39]. Further clarification in the literature among Caucasians and African Americans investigating opioid-linked mortality regarding chronic pain conditions may benefit in contextualizing the opioid epidemic from an ethnocultural viewpoint [25]. Notable disparities among Caucasians and African Americans exist regarding opioid access, addiction, and mortality, prompting discussion of medical treatment options.

#### 4.1.2. Medical Treatment for OUD

Methadone and buprenorphine are common treatments for OUD, and prescribing practices for each largely differ based on race and ethnicity. For instance, buprenorphine is safer than methadone but more commonly distributed in Caucasian communities, while methadone is more commonly distributed to African Americans [7,8,9,40,41]. Findings by Amiri and colleagues [42] suggest that African Americans living near buprenorphine and methadone providers may still choose the lower-tier clinic, potentially secondary to discrepancies in health insurance or perceptions of provider bias. Similarly, Andraka-Christou [7] notes that Medicaid is more prevalently used in the African American population but less commonly accepted by substance use disorder treatment facilities in African American communities. Methadone and buprenorphine both intend to treat opioid-induced hyperalgesia (OIH), a significant decrease in pain-pressure threshold secondary to opioid exposure, through different mechanisms. In managing OIH, buprenorphine is more successful at managing associated symptoms of depression, anxiety, and stress, while methadone may have more cardiac side effects, a more intensive treatment regimen, and is more lethal if not taken as directed [7,42,43,44]. Experiences with methadone, disparities in access, and perceptions of provider bias may be contributing factors to fewer African Americans undertaking treatment for OUD [8,45]. When considering Engel’s biopsychosocial model [26], buprenorphine’s role in ameliorating adverse mental health phenomena may be a significant factor in alleviating psychosocial strain during recovery.

From a biopsychosocial framework, buprenorphine may be more effective in managing the psychosocial aspects of the condition, improving patient quality of life [26,43]. Although patients treated with buprenorphine tend to have lower stress and anxiety, retention rates in either ethnicity among either medication are not statistically significant. However, studies disagree on levels of social support among buprenorphine and methadone users, which would be significantly important as social support likely influences outcomes per the ecological systems theory [9,25,46,47]. This poses a gap in contemporary literature. The qualitative perspectives of each population would be beneficial in highlighting the importance of social support and OUD treatment, contextualizing these findings within biopsychosocial and ecological system models [25,26]. Ultimately, an understanding of treatment methodologies targeting both peripheral and central processing mechanisms of chronic pain via a team of providers emphasizing the biopsychosocial model may assist in the short-term and long-term success rate of opioid reduction [26,41,43,48,49]. Understanding underlying ethnic disparities in access and treatment methodologies provides a greater psychosocial context to each population, which is essential considering the role of neuroplastic contributions in chronic pain conditions [20] (Table 1).

### 4.2. Nociplastic Pain Considerations

The general systems theory emphasizes the value of understanding underlying subsystems, as their interrelationships significantly affect each other and contribute to the overarching problem [24]. Therefore, an understanding of the problem of chronic pain and opioid use disorder in each ethnicity necessitates a review of neuroplastic changes (e.g., a component of the biological system) in chronic pain and OUD and examines its relevance to Caucasian and African American ethnocultural perspectives of pain. Nociplastic pain research has grown significantly in the last 20 years, emphasizing the complexities of central processing mechanisms and the parallels of pain to addiction. Nociplastic pain occurs secondary to maladaptive neuroplasticity, largely correlating to the onset and persistence of OUD [49,50,51]. With persisting pain, changes in neuroplasticity heighten central sensitivity, often resulting in pain that fluctuates disproportionately to actual tissue damage [20,50,51,52]. In the context of the below information, it is important to recognize that pain is an amalgamation of peripheral nociceptive, neurogenic, and nociplastic pain phenotypes. The levels of contribution of a singular phenotype vary due to numerous factors, including but not limited to mechanism of injury, duration of injury, patient sociocultural stressors, patient health beliefs, and the environment in which the injury occurred. Subsequently, nociplastic pain tends to be associated with chronic pain conditions that are difficult to treat secondary to the heavy involvement of biopsychosocial factors [20,49,53,54].

For instance, over half of those with chronic low back pain may also have symptoms consistent with anxiety, depression, or substance use disorders, and many of these symptoms precede the onset of the low back pain. Therefore, psychosocial stressors may be a predisposing and perpetuating factor to the development of chronic low back pain, and many of the above-mentioned sociocultural discrepancies may contribute to the pain experience through the biopsychosocial model [4,14,15,16,17,26,55,56,57]. Similarly, contemporary research suggests that knee osteoarthritis is significantly linked to neuroplastic changes in the spinal cord and brain [58,59,60]. For instance, Liu and colleagues [60] report that individuals with knee osteoarthritis may have increased left parahippocampal and caudal anterior cingulate cortex volume, both potentially affecting individuals’ perception and emotional response to pain. These changes may be potential contributors to pain chronicity and movement apprehension [60]. Both of these pathologies are especially prevalent in those suffering from chronic pain, and understanding the ethnocultural specifics underlying pain and functional disability necessitates a summary of the neuroplastic changes associated with the transition to chronic pain [14,48,55,58,59,60]. Therefore, the sections below will investigate neuroplastic changes in the transition from acute to chronic pain, the neurophysiologic relationship between pain and addiction, and ethnocultural pain perspectives in creating an individualized pain experience.

#### 4.2.1. Neuroplasticity in the Transition from Acute to Chronic Pain

Whether an injury of insidious or traumatic onset, the initial days of the acute condition are characterized by C-fiber stimulus (i.e., threat) from the periphery barraging the interneuron (i.e., an inhibitory neuron that acts as a gatekeeper) at the level of the spinal cord dorsal horns. After stimulating wide-ranging dynamic neurons that lead to the brain, acute pain is often ameliorated through descending pathways via endogenous opioid release, reducing the pain experience [20,61,62]. Usually, pain wanes as tissue healing occurs. However, in the transition to chronic pain, prolonged C-fiber stimulus results in maladaptations to the interneuron and second-order sensory neurons, markedly reducing the effectiveness of gatekeeping sensory input. Over time, light touch sensation may also stimulate nociceptive-specific afferent tracks, interpreting light touch as pain (i.e., allodynia). At this time, the brain receives inputs from both C-fibers and A-beta fibers, resulting in increased ambiguity of the sensation to the brain (e.g., laterality discrimination deficits). Additionally, the endogenous opioid output mechanism diminishes, often increasing the pain experience. The level of interaction and neuroplastic changes at the cord is significant in the transition from acute to chronic pain, and the resulting alterations to brain neuroplasticity further elucidate the complexities of nociplastic pain [20,51,61,63,64].

#### 4.2.2. The Pain Neuromatrix

The pain neuromatrix is significant, as psychosocial experiences and context can modulate the pain experience, and its existence is debated in the academic literature secondary to relative novelty and complexity. However, contemporary literature strongly supports the existence of the pain neuromatrix (e.g., through functional magnetic resonance imaging (fMRI) studies of individuals in pain) [14,20,48,51,61,62,63,64,65]. After passing the interneuron, a stimulus indicating a threat to the system is analyzed in an individual’s pain neuromatrix, comprising the amygdala, primary somatosensory cortex, hippocampus, anterior cingulate cortex, primary motor cortex, thalamus, hypothalamus, prefrontal cortex, cerebellum, and more. With each region participating in the pain response, the efficiency of their inherent primary and secondary functions is reduced, resulting in impaired movement coordination, difficulty with concentration, increased susceptibility to mental health problems, and more. Prolonged stimulus and resulting neuroplasticity of the pain neuromatrix may result in a significant shift in information processing, including but not limited to altered emotional factors and impaired cortical representation of the body schema [20,53,54]. With the pain neuromatrix comprising a sizeable neural network spanning many brain regions, chronic pain can be significantly amplified or reduced by the presence of fear, uncertainty, and stress, which broadly vary among different ethnicities [14,48,53]. PNE fundamentally seeks to address these factors to reduce pain catastrophizing and faulty pain cognitions, potentially influencing a patient’s opioid dependence [20,57].

#### 4.2.3. Neurophysiologic Similarities in Pain and Addiction

Addiction and nociplastic pain share numerous neurobiological similarities, and nociplastic pain may contribute to the progression of OUD and increased odds of relapse. A notable neurologic mechanism is the maladaptation of the brain reward pathway in the presence of persisting pain, increasing an individual’s susceptibility to addiction [41,49,54]. Persisting chronic pain may result in vulnerability to opioid abuse secondary to dysregulation of the endogenous opioid mechanism [41]. From a general systems approach, dysregulation of the endogenous opioid mechanism may facilitate more pain, altered mood, increased urge to consume opioids, altered nutritional attainment, altered immune function, and more—a consequence of system anamorphosis [24,41].

Similarly, chronic pain and drug addiction may facilitate neuroplasticity of the prefrontal cortex and influence decision making, compulsivity, and pain processing [66,67]. The chronification of pain and OUD may be facilitated by projections to the periaqueductal gray (PAG), amygdala, hippocampus, and more to or from the prefrontal cortex [67,68,69]. The PAG is essential to the functioning of the descending pain modulation system, and alteration to this brain area may alter both pain modulation and autonomic nervous system activity, heightening pain perception [68,69]. Similarly, the PAG is postulated to be a perpetuating factor in opioid use by contributing to the OIH during withdrawal [70]. The amygdala and hippocampus exhibit significant increases in neural connectivity to the prefrontal cortex in the presence of chronic pain, which likely facilitates movement apprehension and pain catastrophizing. This hyperconnectivity reportedly diminishes with rehabilitative intervention. Consequently, neuroplasticity in both organs in chronic pain may act as a perpetuating factor through a psychosocial lens [50,67,71]. Regarding OUD, similar changes in the amygdala and hippocampus increase opioid craving and may produce adverse affective experiences, resulting in a stronger sympathetic response that increases relapse rate [72,73,74].

Additionally, individuals with persisting pain often have a reduction in γ-aminobutyric acid (GABA) levels. Reductions in GABA may be associated with compulsive behaviors and depression. From a general systems approach, a reduction in mental well-being and decreased inhibition of the pain experience may be contributing factors to the development of OUD [67,75,76]. An important intersection between pain and addiction exists and is complicated by additional psychological stressors. PNE’s emphasis on the biopsychosocial model targets the underlying psychosocial implications of chronic pain as a potential method of reducing opioid dependency [20,41].

#### 4.2.4. Ethnocultural Perspectives

Neurophysiological changes from acute to chronic pain are influenced by pain-related beliefs that may vary based on sociocultural context, directly influencing pain severity, irritability, and chronification [14,77]. Cultures have markedly different views on pain, comprising the macrosystem of the ecological systems theory and significantly impacting individual development [25]. Some of these cultural perspectives may be influenced by underlying factors in the development of pain and disease, including but not limited to ethnic discrepancies in weight, mobility, heart disease, depression, and diabetes [78]. These factors may functionally serve as maladaptive physical and mental pain amplifiers or inhibitors, especially in the context of nociplastic pain, where psychosocial and neuroimmune influences are significant [20].

Because those with chronic pain may be neurophysiologically sensitive to psychosocial stressors, evaluation and beliefs regarding persisting pain likely modulate the pain experience [14,55,62]. African Americans are more likely to employ emotion- and problem-focused coping strategies such as stoicism, catastrophizing, and spirituality compared to Caucasians, and perceived bias and discrimination among African Americans regarding healthcare providers are positively associated with worse pain outcomes [4,15,17,77,79]. On the contrary, Orhan and colleagues [77] report that Caucasians may be more likely to implement adaptive coping strategies that include trying to ignore or control pain, potentially resulting in increased self-efficacy and improved perceived locus of control. Perceived locus of control plays a crucial role in chronic pain, as individuals who feel less control over their circumstances report higher pain severity [80]. Rigg and colleagues [18] report that African Americans who are misusing opioids are more likely to use them to self-manage pain rather than for recreational purposes. This may be secondary to increased distrust in the medical system, which may be facilitated by provider bias, previously poor experiences with medical professionals, and patient trust in the provider [4,11,14,18]. Specifically, Greenberg and colleagues [81] highlight that African Americans may experience lower quality care. This discrepancy may have behavioral implications by reinforcing the inclination that an individual needs to self-manage symptoms without appropriate medical supervision [18].

Using fMRI, researchers noted significantly higher activity in frontostriatal regions that are associated with pain valuation, regulation, and chronification in the presence of thermal stimulus in African American versus Caucasian samples. These same regions are also associated with pain catastrophization and anticipatory anxiety, worsening if the participant has less trust in the provider. In this case, researchers used a 30-year-old White male provider, and African American participants noted significantly decreased trust compared to Caucasian participants [14]. Ethnocultural perspectives of pain may differ by the participant’s trust in the provider, resulting in statistically significant frontostriatal activity and potentially altering the pain experience.

Secondary to ethnic perspectives and health beliefs, disparities such as healthcare access, care quality, and socioeconomic status, alongside other social determinants of health, may play a significant role in pain valuation [15,16]. Chronic pain may result in increased pain catastrophizing and disability in the African American population, compounded by differing views of healthcare providers regarding bias [4,14,17]. Ultimately, pain is individualistic, and an individual’s surrounding ecological systems largely shape individual development. For instance, an individual’s pain beliefs are shaped by experiences in their immediate environment, witnessed and learned events in their indirect environment, and sociocultural values [20,25,53,54]. These pain beliefs may amplify or inhibit the pain response [14,29,48,57]. Understanding the role of cultural stressors regarding pain (e.g., pain beliefs and trust in the provider) is essential for optimal intervention deployment, such as PNE [14,20,48]. However, while beliefs regarding the evaluation and management of pain may differ culturally, the complexities of the pain neuromatrix reveal that each individual will have a unique pain response [14,20,48,51,61,62,63,64,65]. Therefore, analyzing ethnic discrepancies in pain beliefs should not supersede an individual’s presentation, as each individual will experience a markedly different pain response based on a plethora of biopsychosocial factors [62,80]. Impaired psychosocial well-being is especially prevalent in those misusing opioid medications for chronic pain, potentially perpetuating or exacerbating the chronic pain experience and facilitating further opioid misuse [18,48,65]. Ultimately, contemporary sources suggest ethnic disparities exist and influence chronic pain through extranociceptive mechanisms. This phenomenon results in unique pain evaluation within the pain neuromatrix, including the pain stimulus alongside psychosocial factors [14,20,25,53,54] (Table 2).

### 4.3. Pain Neuroscience Education

PNE utilizes the biopsychosocial model, fundamentally grounded in general systems theory, to treat nociplastic pain conditions through reduced faulty pain cognitions. An individual’s belief systems play a significant role in the success of PNE, with biopsychosocial intervention often challenging predominantly biomedical belief systems [20,24,26,29,30,57,82]. Indicators for success in PNE are discussed below to highlight factors associated with clinical expectations. For delivery strategies, complex topics like peripheral and central processing mechanisms are explained in more straightforward metaphors that are effective in reducing faulty pain cognitions [20,21,22,29,65]. Physical therapy intervention is inherently multimodal, and research suggests that PNE is more effective when used as an adjunct intervention to other physical therapy interventions, specifically those that emphasize active movement [30,57,82,83,84,85,86,87]. Alongside delivery strategies, indicators for success are investigated to elucidate PNE expectations and implementation.

#### 4.3.1. Delivery Strategies

PNE is often delivered with or without additional intervention through stories and metaphors to effectively educate patients on the complicated pain process. Numerous metaphors exist, with more popular metaphors including the alarm system, nerve sensors, nosy neighbors, and the wet brain [29,57]. Comparing nociception and central or peripheral sensitivity to an alarm system emphasizes the purpose of pain as a protective mechanism, looking to reduce faulty cognitions associated with tissue damage. The nerve sensor story, a metaphor for peripheral sensitivity through ion channel expression, elucidates the reasoning as to why pain conditions may increase in the presence of stress or temperature changes, improving patient understanding of seemingly ambiguous phenomena and ideally improving their sense of safety. In nociplastic pain mechanisms, pain may feel like it is spreading to vast parts of the body. The nosy neighbor metaphor is a simplified introduction to neuroplasticity and peripheral sensitization for the patient, clarifying an unclear phenomenon. Lastly, the wet brain discusses a simplified version of the endogenous opioid mechanism for pain control, empowering the patient by providing examples (e.g., exercise, comedy shows, etc.) of activities that stimulate endogenous opioid release [29].

Contemporary researchers interested in pain neuroscience in the field of physical therapy agree that PNE is best used in the context of individually tailored physical therapy intervention (e.g., manual therapy, exercise therapy, neurodynamics, etc.) [20,21,83]. The goal of PNE is to empower the patient and reduce faulty pain cognitions, which may present in the form of kinesiophobia and movement apprehension. Ameliorating a contributing factor to fear of movement may permit increased aerobic exercise, allowing the patient to gradually increase participation in personally meaningful activities. This increase in participation may decrease stress, fear, and anxiety, calming central processing mechanisms and ameliorating the pain experience [20,23,29,83].

However, allodynia or hyperalgesia may sometimes result in poor tolerance to traditional therapeutic engagement, necessitating the utilization of graded motor imagery (GMI) to advance the patient clinically. In patients with significant allodynia or hyperalgesia who do not tolerate movement, GMI has become a novel treatment approach to progress these patients toward the level of more traditional physical therapy [20,29,83]. In the past 5 years, the literature surrounding the use of GMI has grown for nociplastic conditions, especially complex regional pain syndrome. This treatment ideology strives to facilitate beneficial neuroplasticity by reorganizing the sensory and motor cortices and other components of the pain neuromatrix. GMI is delivered sequentially in three levels: laterality discrimination, explicit motor imagery, and mirror therapy [88,89,90,91]. As GMI is fundamentally grounded in alleviating maladaptive neuroplastic changes, it is an excellent component of a multimodal treatment plan alongside PNE for complex nociplastic conditions. Adding PNE with GMI improves pain cognitions and applies newly learned information to sensory input and early mobility, integrating feelings of safety necessary for appropriate clinical progression [20,88,91]. PNE is likely best used as an adjunct to other physical therapy interventions, which may range from GMI for high-severity and high-irritability pain patients to traditional exercise intervention for others [30,57,82,83,84,85,86,87,88,89,90,91]. Overall, the best outcomes may occur when the information found in PNE emphasizes the development of self-efficacy and is applied to functional mobility or sensory input for effective graded exposure strategies [23,83].

#### 4.3.2. Indicators for Success

Success with PNE refers to reducing opioid dependency through the reduction of faulty pain cognitions. As nociplastic pain can persist in the absence of tissue damage, PNE challenges biomedical models in the presence of chronic pain and challenges the individual’s belief system with the concept that chronic pain is inherently biopsychosocial, serving a protective role [82]. Therefore, patients may benefit more if they are at least within the contemplative phase of the transtheoretical model, as their underlying intrinsic motivation and behavioral factors will affect the success of the learning process [20,23].

Duration of the education session likely has a significant impact, with shorter sessions reducing cognitive fatigue and optimizing recall. Qualitative studies show mostly favorable results when measuring patient perception regarding PNE implementation, and readiness to change may be a predominant factor in the other studies [92,93,94,95]. Participant pain beliefs and the effectiveness of the communication strategies of the individual delivering PNE were major themes affecting patient perception. For example, Oosterhaven and colleagues [95] implemented education sessions, each over an hour long, and found that their chronic pain sample largely could not remember the information provided and poorly applied it to their present condition. On the contrary, subjects of other studies that utilized shorter education sessions noted a paradigm shift in their understanding of pain, resulting in improved self-efficacy regarding self-management strategies [92,93,94]. Therefore, a worthwhile consideration in improving the effectiveness of PNE may be shorter duration education sessions, alongside other factors like nocebo.

As pain is influenced by psychosocial factors, nocebo from other providers emphasizing biomedical language can result in a marked increase in pain catastrophization, worsening symptoms, and disability [14,20,48,96]. Nocebo refers to maladaptive neurobiological changes secondary to actual or perceived harm and can result from negative provider–patient interactions, threatening medical models in the clinic, and previously learned experiences. When using fMRI, studies show increased PAG activity when told that an intervention may have painful side effects, resulting in an increased pain response [48]. Additionally, emphasis on terms like “bone-on-bone”, lack of interpretation of imaging findings, and medical models that show pathology may also provoke fear and uncertainty, leading patients to believe that their pain will persist and that conservative treatment methodologies will be ineffective [27,28]. Lastly, previously learned experiences (i.e., a particular movement provokes pain) may create anticipatory expectations, facilitating or amplifying the pain response [48]. The individual’s prior clinical interactions and learned experiences may result in furthering faulty pain cognitions, reducing mobility, and exacerbating pain presentations. Consequently, considerations for success with PNE in nociplastic pain conditions may also include acknowledging an individual’s healthcare history, challenging biomedical beliefs, and reducing inaccurate understandings of pain.

One of the most effective strategies in improving the intrinsic motivation of learners is targeting their self-efficacy [23]. First, self-efficacy can apply to the learning process itself. Individuals who are motivated by the educator to continue learning about pain neuroscience may benefit from an increased understanding of their pain experience, facilitating a reduction in faulty pain cognitions and potentially improving outcomes [23,29]. Second, self-efficacy may facilitate an improvement in an individual’s locus of control over their situation, which may be a protective factor against chronic pain severity [23,29,77,80]. However, patient-provider interactions lacking an emphasis on psychosocial well-being and foundational pain neuroscience principles may also have deleterious outcomes in the patient’s self-efficacy, reducing participation in future programs [27,28,48].

Lastly, a patient needs hope and trust in the provider. A lack of empowerment may result in a lack of participation in PNE or exercises to improve their condition. The role of empowerment may differ among ethnicities, as a research study using fMRI noted higher frontostriatal activity in African Americans than Caucasians when being treated by a White provider [14]. Unfortunately, many nociplastic pain patients have failed numerous medical treatment types. Educating a patient on pain, optimizing delivery strategies, and improving self-efficacy regarding management strategies are excellent ways to instill hope [20,29] (Table 3).

## 5. The Research Gap

PNE research in the last 10 years lacks integration of various factors, such as readiness to change, age, and ethnicity. Research should incorporate readiness-to-change models, such as the transtheoretical and health belief models, into PNE implementation. Furthermore, existing studies have not adequately analyzed variations based on age groups, particularly considering generational response differences. Contemporary PNE research elucidates a lack of experimental designs regarding PNE intervention and outcomes among different ethnicities [21]. Understanding the temporal context, generational influences, and ethnocultural influences on pain in the context of PNE will significantly enhance academic knowledge regarding this intervention.

### 5.1. Readiness to Change

Recent studies fail to differentiate patients by their health beliefs in the context of PNE, resulting in controversy regarding prognostication [21]. A patient who lacks the intention to change may have a markedly different response to information that challenges biomedical health beliefs than a patient who intends to change. Intent to change and underlying beliefs of benefit or severity are staple domains of the transtheoretical model and health belief model, and contemporary PNE research would benefit from incorporating these models in experimental study designs with standardized outcome measures (e.g., Tampa Bay Scale of Kinesiophobia, Central Sensitization Inventory) to identify patients who may best receive PNE intervention [97,98]. Investigating the relationship of the stages of change of the transtheoretical model or the underlying domains of the health belief model to PNE implementation may provide clinically meaningful information regarding PNE indicators for success [99,100].

### 5.2. Age Group Analysis

Age group analysis may increase specificity in understanding PNE, as research lacks information regarding differing generational perspectives of PNE and utilization of PNE in adolescent populations. Relevant research investigating generational views of pain within the context of pain neuroscience is performed by Zimney and colleagues [101]. This study utilizes a cross-sectional descriptive survey, so further research utilizing a more rigorous, experimental design (e.g., randomized controlled trial) that investigates generational differences of PNE on pain or pain cognitions fills a notable gap in existing literature. Implementing age group analysis in the context of PNE may elucidate further information regarding PNE indicators for success and delivery strategies, especially if combined with further research regarding ethnicity and PNE.

### 5.3. Ethnic Group Analysis

Studies examining African American and Caucasian perspectives of pain and frontostriatal activity lay the framework for potential variation in central processing mechanisms between ethnicities [14,15,16,17]. Building off the work by Colloca [48] and Losin and colleagues [14], examining frontostriatal activity via fMRI with PNE implementation in individuals of different ethnicities will be beneficial in providing an ethnocultural context to PNE. Since Losin and colleagues [14] note that provider buy-in affects pain (i.e., resulting in increased frontostriatal activity), both qualitatively and quantitatively, investigating the impact of providers of differing ethnicities on PNE and patient frontostriatal activity may be beneficial in understanding indicators for success and delivery strategies of PNE. PNE’s impact on brain activity can be observed by fMRI, resulting in an excellent avenue for investigating ethnocultural pain processing [14,48]. Because each person’s pain experience is individualistic per the complexities of the pain neuromatrix, qualitative studies that compare patient experience and their fMRI findings may be beneficial and provide valuable context to the alterations in brain activity. Additionally, randomized controlled trials investigating the effectiveness of PNE in reducing pain catastrophizing should compare results among ethnicities, secondary to the differences in health beliefs and sociocultural context [4,14,17].

### 5.4. Investigating Ethnocultural Differences in the Combination of GMI and PNE

Since fMRI data show variation in mesolimbic and frontostriatal activity in the ethnocultural perception of threat, the investigation into the combination of GMI and PNE will benefit the development of academic literature in these fields [14,48]. Fundamentally, GMI and PNE encourage beneficial neuroplastic changes through graded exposure strategies to counteract maladaptive changes to regions of the brain comprising the pain neuromatrix. GMI has been primarily studied in phantom limb pain and complex regional pain syndrome; however, studies regarding GMI in other chronic pain conditions seem to be increasing [88,90,91]. Experimental research designs (e.g., double-blinded randomized controlled trials) investigating GMI and PNE as a multimodal treatment approach for under-researched chronic conditions would greatly benefit contemporary clinical practice, as many clinicians struggle to manage the nuances of this pain phenotype [65]. Additionally, longitudinal cohort studies assessing the carryover of GMI and PNE on patient functional mobility and health beliefs may be beneficial to provide a greater temporal context to these interventions.

## 6. Conclusions

PNE, as an adjunct to other physical therapy interventions, is a promising tool for reducing opioid dependence, as it treats the patient from a biopsychosocial perspective and emphasizes personally meaningful activities. Nociplastic pain is likely underappreciated clinically in chronic conditions, as providers often conceptualize pathology in the biomedical realm, which may amplify the pain response [48]. Furthermore, appropriate delivery of PNE and additional intervention should emphasize the underlying differences in ethnic perspectives of pain, as frontostriatal activity may increase with a lack of trust in the provider and result in increased activity of notable pain centers [14]. Therefore, a biopsychosocial approach is essential, especially given the rise in opioid use and mortality in the last few decades [31,32,33]. Implementing PNE to reduce faulty pain cognitions often prevalent in nociplastic pain (e.g., pain catastrophization) may be an option in combating the opioid epidemic [48,65,81]. Future research should consider further investigation of PNE regarding temporal context (i.e., patient readiness to change), generational context, ethnic context, and graded motor imagery for clinical implementation and to advance contemporary academic knowledge.

## Figures and Tables

**Table 1:** Key points: opioid dependency.

	Summary Statements
1	Caucasians receive higher amounts of opioid medications than African Americans, contributing to higher rates of OUD and opioid-related mortality [4,12,31,32,33].
2	While reporting higher pain levels on average, African Americans receive less opioid pain medication. Likewise, African Americans experience a notably higher rate of increase in polysubstance overdoses (e.g., opioids plus cocaine) and are more likely to use opioids for the self-treatment of chronic pain [5,7,10,11,12,13,18,36,37,38,39].
3	Buprenorphine is more effective than methadone at addressing psychosocial symptoms (e.g., anxiety and depression) but is less available to African Americans [7,42,43,44].

**Table 2:** Key points: nociplastic pain considerations.

	Summary Statements
1	Neuroplastic changes can occur in chronic pain syndromes, resulting in pain that is often disproportionate to tissue damage [14,20,49,53,54,55,62,67,68,69].
2	Pain is individualistic secondary to the complexities of the pain neuromatrix [14,20,48,51,61,62,63,64,65].
3	Chronic pain and drug addiction share neurophysiologic similarities, including descending output dysregulation and neuroplastic changes in connectivity to and from the prefrontal cortex [41,49,54,66,67,68,69,70,71,72,73,74,75,76].
4	Social and ethnocultural factors may modulate the pain experience [14,15,16,17,18,55,62,77,79,80,81].
5	Regarding pain coping strategies, African Americans may be more likely to use emotion and problem-focused coping strategies (e.g., stoicism, catastrophization, spirituality), and Caucasians may be more likely to use adaptive-focused coping strategies (e.g., ignoring or trying to control pain) [4,15,17,79,80].

**Table 3:** Key points: pain neuroscience education.

	Summary Statements
1	PNE is grounded in the biopsychosocial model to reduce faulty pain cognitions and movement apprehension through facilitating the understanding that pain can be a protective, centrally mediated experience [20,24,26,29,30,57,82].
2	Metaphors and stories are used to simplify complex pain concepts to improve relatability to patients [29,57].
3	PNE sessions that are shorter and more focused may have increased retention and cognitive engagement [92,93,94,95].
4	In physical therapy, PNE as an adjunct to traditional intervention is more effective than PNE alone [30,57,82,83,84,85,86,87].
5	The combination of PNE and GMI may be clinically useful in those with severe hyperalgesia or allodynia [88,89,90,91].
6	Nocebo from negative patient–provider interactions, threatening medical models, and learned experiences may be deleterious to care goals [14,20,27,28,48,96].

## Data Availability

No new data were created or analyzed in this study. Data sharing is not applicable to this article.
